# Emerging Role of Renal Sympathetic Denervation as an Adjunct Therapy to Atrial Fibrillation Ablation

**DOI:** 10.31083/j.rcm2504122

**Published:** 2024-03-28

**Authors:** Krittapoom Akrawinthawong, Takumi Yamada

**Affiliations:** ^1^Section of Cardiac Electrophysiology, University of Oklahoma Health Science Center, Oklahoma City, OK 73104, USA; ^2^Section of Cardiac Electrophysiology, Cardiovascular Division, University of Minnesota, Minneapolis, MN 55455, USA

**Keywords:** ablation, atrial fibrillation, renal denervation

## Abstract

The central anatomical locus in the context of atrial fibrillation (AF) ablation has been the pulmonary veins. Despite the attainment of a modest long-term success rate through pulmonary vein isolation (PVI), the pursuit of achieving a therapeutic efficacy nearing a definitive cure has spurred an investigation into alternative strategies and anatomical loci beyond the pulmonary veins. Despite extensive exploration, none of these alternative targets have succeeded in establishing themselves as routine ablation sites comparable to the pulmonary veins. Consequently, there exists an imperative for further inquiry and refinement of ablation strategies to propel advancements within the domain of AF ablation, thereby augmenting patient outcomes. Simultaneously, the examination of the autonomic system’s role in AF pathophysiology introduces an additional ablation target aimed at rectifying sympathovagal imbalance. This discourse presents a contemporary review of renal denervation (RDN) as an emergent and auspicious technique poised to complement PVI, thereby contributing substantively to the augmentation of long-term success within the ambit of AF rhythm-control strategies.

## 1. Introduction

The pulmonary veins were revealed to be the major source of atrial fibrillation (AF) two decades ago [1, 2, 3]. Since then, pulmonary vein isolation (PVI) has been established as a cornerstone in catheter ablation of AF. In cases of paroxysmal AF, the long-term success rate of catheter ablation has reached its pinnacle at 70–80% [4]. However, for persistent AF, this figure diminishes to a modest 60–70% [5]. Therefore, catheter ablations beyond PVI such as several ablation lesion sets targeting various anatomical structures of the atria other than the pulmonary veins, along with techniques to identify individual extra-pulmonary vein triggers of AF, have been developed in an effort to enhance the rate of AF freedom [6, 7, 8, 9]. Regrettably, none of these approaches have attained the status of a standard of care, unlike PVI. Hence, further research and refinement of strategies are imperative to advance the field of AF ablation and improve patient outcomes.

This article presents a contemporary review encompassing the rationale behind renal denervation (RDN) for AF management, the various tools involved, the efficacy and safety of the procedure, and the supporting evidence from relevant studies for a comprehensive understanding of the potential role of RDN as an adjunct therapy to PVI.

## 2. Role of Autonomic Nervous System in Pathogenesis of AF

Human hearts possess a rich supply of autonomic nerves [10, 11, 12, 13], which govern various cardiac functions. The autonomic nervous system (ANS) has been identified as a key player in the pathophysiology of several arrhythmias, including AF. Modulating this system has demonstrated the potential to alter the electrophysiological properties of cardiac tissue, offering a promising avenue for arrhythmia control. Parasympathetic activation is associated with shortening of the atrial effective refractory period, making the atria susceptible to a reentry [14, 15, 16]. Conversely, stimulation of the sympathetic nervous system enhances intracellular calcium levels, thereby promoting a triggered activity and automaticity [11, 17]. The role of the cardiac ANS in initiating and maintaining AF has been extensively studied, leading to certain ablation approaches, such as an ablation targeting the ganglion plexi [18]. However, the outcomes of AF ablation using this approach have yielded conflicting results [19, 20, 21, 22]. Furthermore, it is crucial to acknowledge that vagal nerves also contain sympathetic components, as evidenced by the immunohistochemical studies [11]. This may explain the ambiguous outcomes observed in AF recurrence following the ganglionic plexus ablation [19] or Vein of Marshall ethanol infusion [23, 24]. The current technology of radiofrequency (RF) energy cannot selectively target either the parasympathetic or sympathetic components of the ANS in the heart, where both components are highly co-localized. A direct autonomic modulation at the cardiac tissue is further complicated by reinnervation and neuroplasticity, which enable nerve sprouting from surviving nerves after the ablation and constant remodeling of these nerves. This complex interplay underscores the need for a continued research and development in order to refine and optimize autonomic modulation as a therapeutic strategy for managing arrhythmias effectively.

The therapeutic effects of sympathetic denervation in controlling arrhythmias have been observed in the extra-cardiac sympathetic system. Adrenergic activation-induced metabolic remodeling plays a pivotal role in the initiation of acute AF and contributes to the progression of AF from a paroxysmal to persistent form [11, 14]. Rebalancing the activation of the ANS holds promise in potentially altering the course of AF progression. Addressing sympathetic activation has proven challenging, as it typically necessitates an invasive surgical procedure at the sympathetic trunk [10, 11, 25, 26]. However, an access to critical regions such as the stellate ganglion and T2-T4 sympathetic ganglion is not easily achievable, and this surgical approach is less favorable due to potential complications and debilitating side effects like hyperhidrosis and postural hypotension. Fortunately, current extracardiac neuromodulation modalities offer more appealing options due to their non or less-invasive nature. These modalities include transcutaneous tragus stimulation [27], stellate ganglion blockage [12, 28], baroreflex receptor therapy [29, 30], and renal denervation (RDN) [31, 32, 33, 34]. By virtue of their non or less-invasive nature, these approaches hold promise for managing sympathetic activation in AF patients more conveniently and with potentially fewer adverse effects.

The central sympathetic outflow is intricately regulated by afferent renal sympathetic signaling mediated through the posterior hypothalamus [11]. The presence of a dense and extensively interconnected network of sympathetic nerve fibers establishes a crucial link between the central nervous system and the kidneys, facilitated through the aortorenal ganglia [35]. This network presents a feasible target for percutaneous catheter ablation, offering a less invasive alternative to surgical approaches. RDN has garnered significant attention as a potential therapeutic approach for managing resistant hypertension [36, 37]. Considering this, RDN holds promise for providing both direct and indirect therapeutic effects on controlling AF, particularly when used in combination with PVI. It is plausible that RDN targeting the renal sympathetic nerves may exert a positive impact on a reduction of AF recurrence [31, 33, 38, 39, 40, 41].

## 3. Tools and Techniques of RDN

The renal sympathetic nerves consist of both afferent and efferent arms, and they are found in the adventitia of the renal arteries [42, 43]. Efferent renal nerve mediates activity of renin-angiotensin-aldosterone system and regulates renal blood flow. Targeting the afferent arm of the renal sympathetic nerves is necessary to modulate the central sympathetic outflow to the peripheral organs. However, due to their close anatomical proximity in the adventitia of the renal arteries, percutaneous RDN procedures typically involve interrupting both arms of the renal sympathetic nerves inevitably. This simultaneous interruption of both arms is a practical challenge in achieving selective modulation but is currently the approach utilized in RDN procedures. Various approaches to ablate the renal nerve have been tested in the past [44], including ultrasound, RF energy, and drug infusion delivered through a catheter [45]. Among these options, percutaneous catheter ablation using RF energy appears to be the most feasible and widely used method. The procedure follows a workflow similar to a routine cardiac ablation for arrhythmia treatment.

**Fig. 1.**
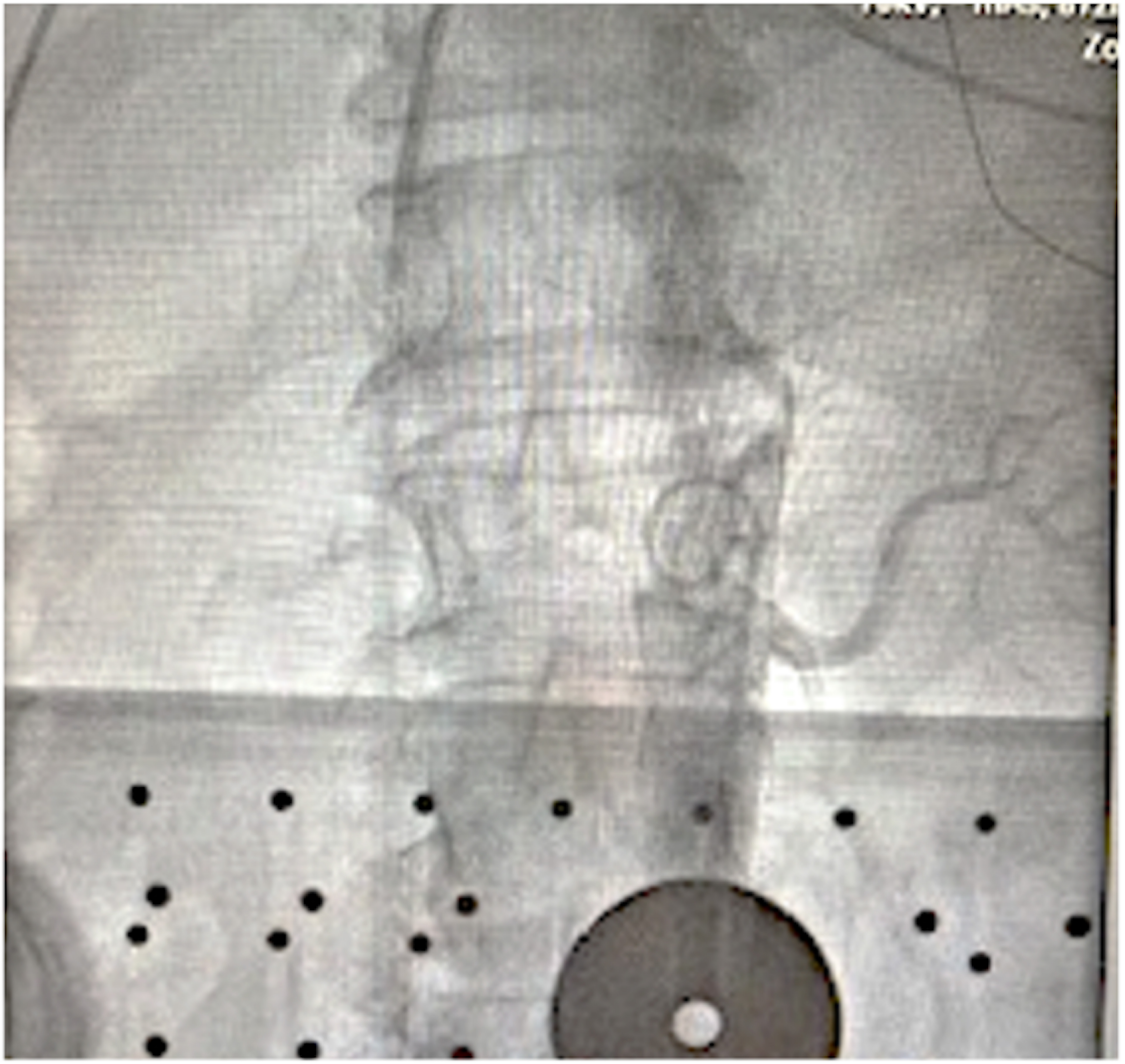
**Abdominal aortography with a pigtail catheter to identify the renal artery**. A pigtail catheter is usually positioned at the vertebra level T1-2 to perform an aortography with a power injector to identify the orifice of bilateral renal arteries and their branches.

**Fig. 2.**
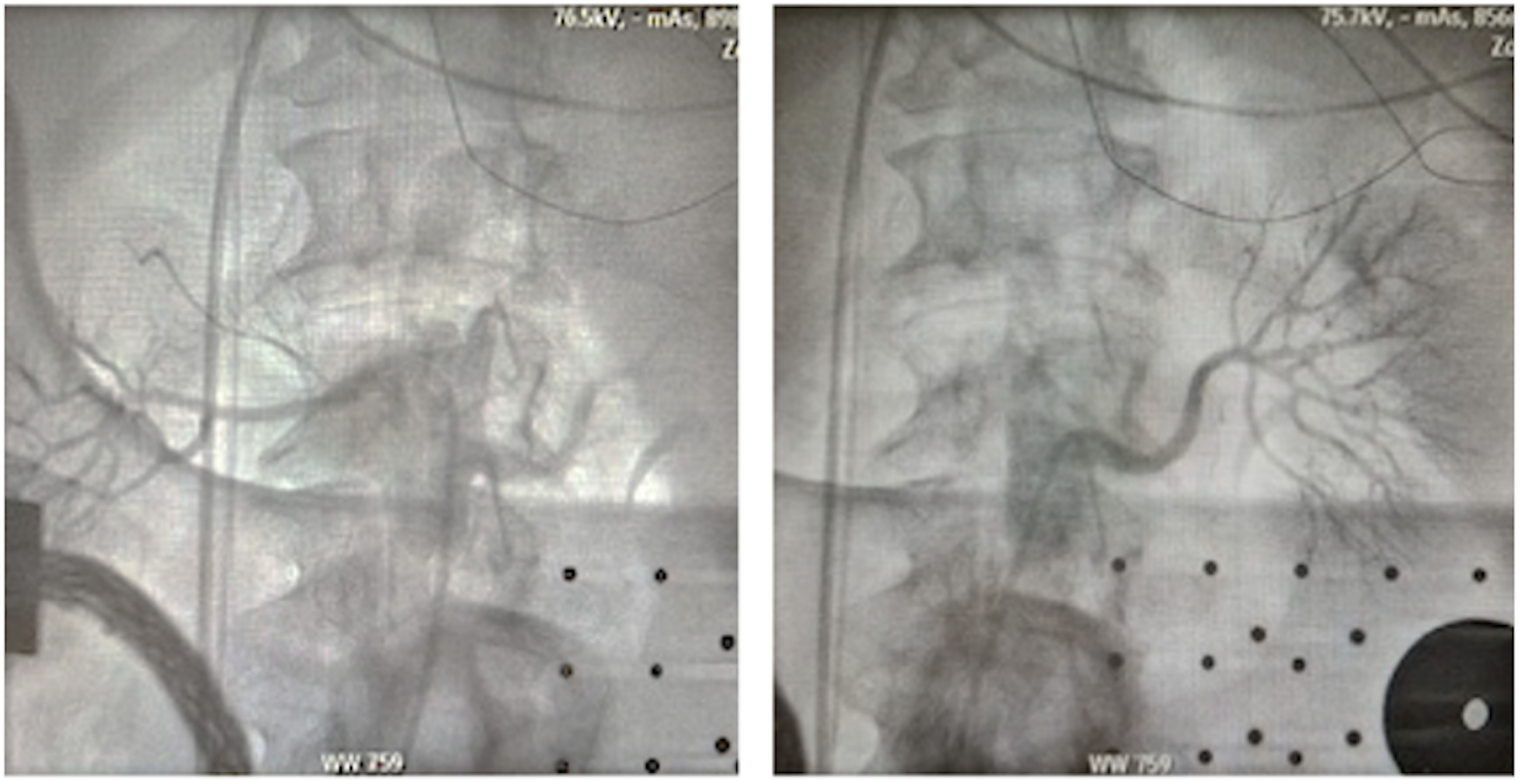
**Selective angiography of the right (left panel) and left (right panel) renal artery**. This step can be performed with various types of diagnostic catheter (LIMA catheter, MP catheter, or RDC catheter). LIMA, left interval mammary; MP, multi-purpose; RDC, renal double curve.

**Fig. 3.**
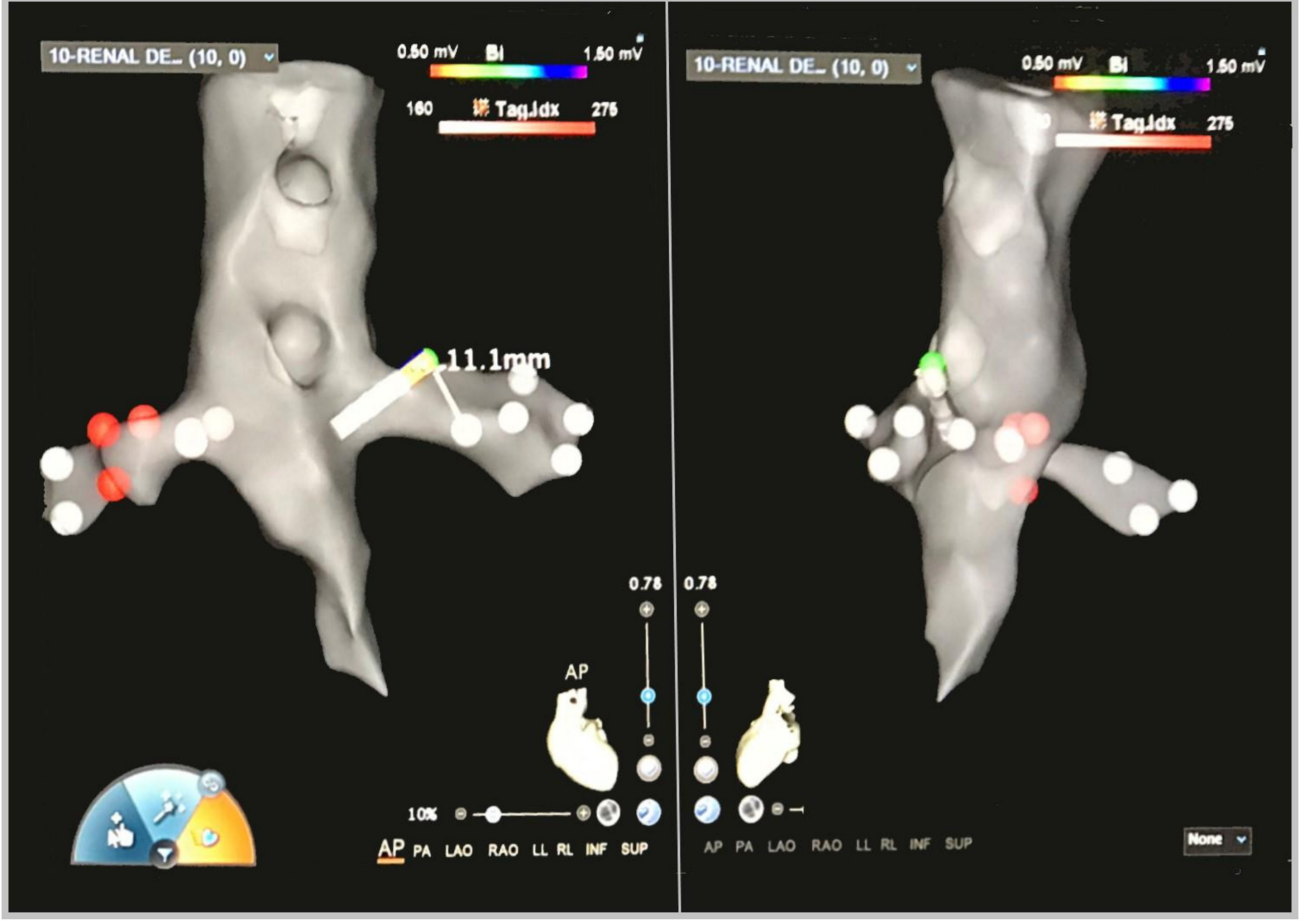
**Fast Anatomical Map of bilateral renal arteries with ablation’s lesion tags performed by standard unipolar ablation catheter on a three-dimensional anatomical map (CARTO3 mapping system)**. AP, anteroposterior; PA, posteroanterior; LAO, left anterior oblique; RAO, right anterior oblique; LL, left lateral; RL, right lateral; INF, inferior; SUP, superior.

**Fig. 4.**
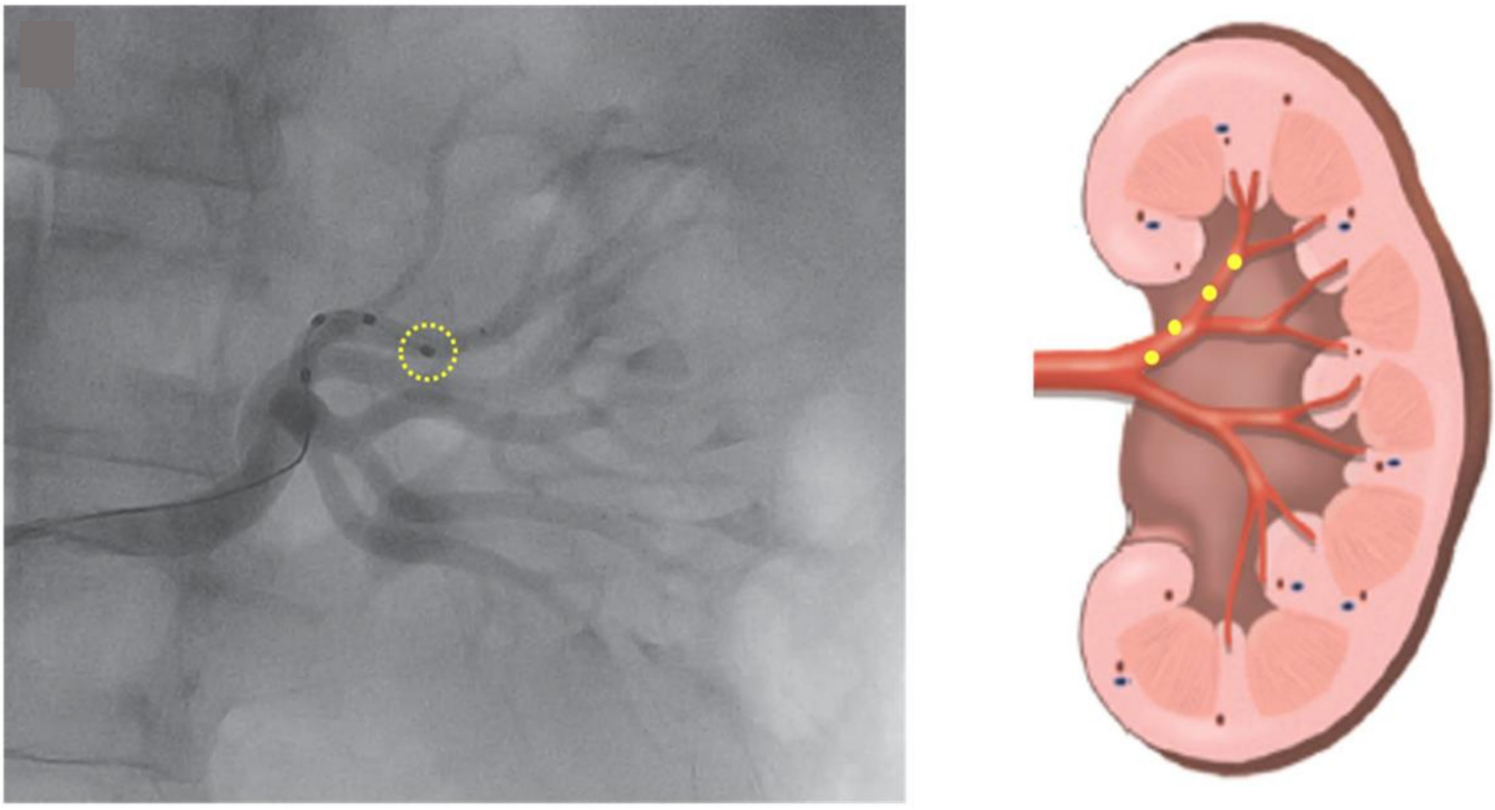
**Renal denervation performed with a multipolar ablation catheter (Medtronic™ Symplicity spiral catheter) that was advanced inside the distal branch of the renal artery**. Kiuchi *et al*. [46]; with permission (Fig. 1A in original).

The process of the RDN by RF catheter ablation typically involves an aortogram, which is performed using a pigtail catheter at the L1-2 vertebral level to visualize the renal artery (Fig. 1). Subsequently, a selective renal angiogram can be carried out using a diagnostic catheter (such as a left internal mammary [LIMA] catheter) to precisely identify the target location for the ablation (Fig. 2). To reduce the reliance on fluoroscopy during the procedure, a three-dimensional (3D) anatomical map of the renal artery and aorta can be constructed using impedance systems through a percutaneous catheter approach via the femoral artery (Fig. 3). Once the target site is identified, a conventional ablation catheter is positioned at the distal part of the renal artery to deliver RF energy application (Fig. 4, Ref. [46]). The RF energy is applied in a circumferential fashion while the catheter is withdrawn proximally (Fig. 3). The power settings for RF energy application typically range from 8 to 12 Watts, with each lesion receiving treatment for 1 to 2 minutes. The procedure is performed in the bilateral renal arteries and their branches.

It is worth noting that single electrode catheters are currently less favored for RDN than multi-electrode catheters due to the extensive neural network of small caliber vessels in the distal renal artery, which may not be well accommodated by larger size of catheters. To confirm the therapeutic effect of RDN, a comparison between baseline and post-ablation high-frequency stimulation tests of the afferent renal nerve is conducted. This involves observing the attenuated effect of an increase in blood pressure levels with stimulation, indicating a successful ablation outcome.

In terms of safety, certain criteria must be met to ensure the procedure is performed without complications. Specifically, the length of the renal artery should be at least 20 mm, and the diameter should be at least 4 mm to avoid any arterial damage [47, 48]. This can be estimated angiographically or more precisely measured using intravascular ultrasound imaging. Additionally, care should be taken to avoid any pathologic damage of the renal arteries, such as those displaying stenosis or calcification, as they may present higher risks for complications during the procedure.

**Fig. 5.**
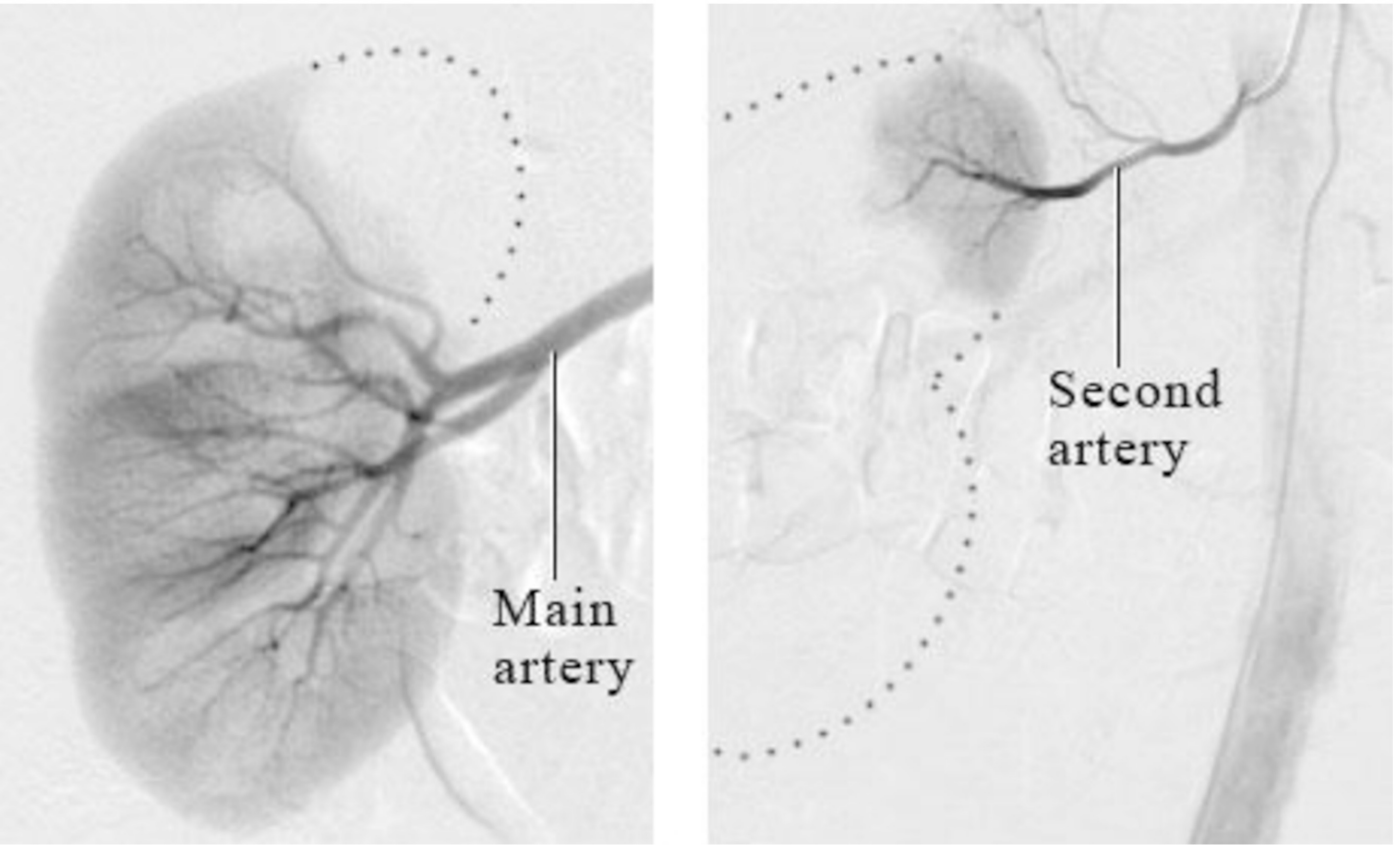
**Partially-unfilled renal parenchyma on renal angiogram suggesting presence of the accessory renal artery (Courtesy image from intermountain medical imaging, Boise, Idaho as a part of Healthwise medical review board)**. References (website): https://www.northshore.org/healthresources/encyclopedia/encyclopedia.aspx?DocumentHwid=zm6032.

Understanding the anatomy of the renal sympathetic nerve distribution is crucial for achieving excellent results of the RDN. The density of sympathetic nerves is highest in the proximal and mid-segments of the renal artery. However, the mean distance of these nerves from the artery is the lowest in the distal segment [42, 43]. Therefore, ablation at the distal part of the renal artery may be more effective approach for the RDN. Accessory arteries, which are also surrounded by sympathetic nerves, can be excellent targets for ablation as well. To ensure that these accessory arteries are not missed during the procedure, it is essential to observe fully-filled renal parenchyma with a contrast (Fig. 5). If any part of the renal parenchyma is unfilled, it may indicate the presence of an accessory artery. The high variation in renal nerve distribution poses a significant challenge in delivering RF energy precisely to reach the target nerves. The depth of the ablation lesion is typically around 2–4 mm [42, 49], which underscores the need for accurately-targeted delivery of RF energy during the procedure.

Considering the complex anatomy and distribution of the renal sympathetic nerves, careful planning and execution of the ablation procedure are critical for achieving successful outcomes in the RDN. Advanced imaging techniques and a thorough understanding of individual patient anatomy are instrumental in guiding the procedure to optimize the therapeutic effects of the RDN in managing AF and other conditions associated with sympathetic nervous system dysregulation.

There are 3 special systems that have been developed for RDN.

**Fig. 6.**
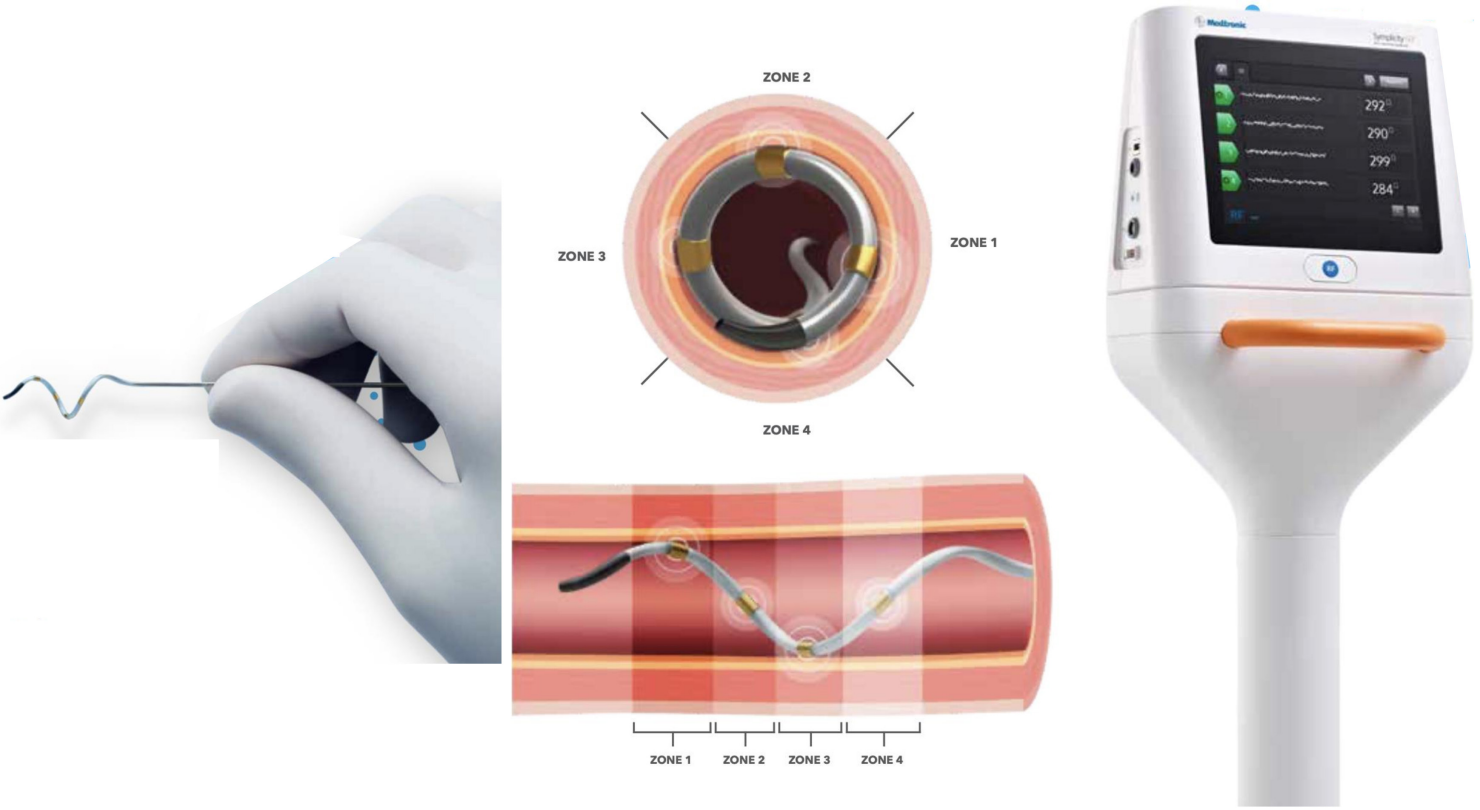
**Medtronic Symplicity Spyral renal denervation system**. A quadripolar catheter with a distal self-expanding array that can be advanced over a 0.014 guidewire. Real-time responsive algorithm on the machine allows for simultaneous ablation of 4-quadrant of the renal artery. Automatically-adjusted power delivery on each electrode is achieved with a real-time impedance and temperature feedback. An individual electrode can be deselected in the unsuitable location for the ablation [Courtesy image from the brochure of Medtronic Symplicity Spyral renal denervation system]. With courtesy from Medtronic.

(1) Medtronic SYMPLICITY system (Fig. 6): The SYMPLICITY system utilizes the Symplicity Spyral™ multi-electrode (quadripolar) RDN catheter, which is a second-generation 6F catheter delivered over a 0.014 inches non-hydrophilic, flexible-tipped wire to the renal artery. The RF application begins distally and gradually moves proximally to the renal artery ostium. Adequate contact of the catheter on the arterial wall is verified by angiographic imaging obtained by a contrast injection from the catheter’s distal end and stable impedance values on each electrode, ensuring appropriate energy delivery throughout at least one respiratory cycle. This system allows for a selective deactivation of any electrode located in the unsuitable anatomy, such as the carina or areas with an arterial disease [50]. This system currently boasts the highest patient enrollment for clinical trials compared to other vendors (Table 1, Ref. [51, 52, 53, 54, 55, 56, 57]).

**Table 1.** **Summarized key randomized controlled trials of RDN in AF patients**.

Study (years)	Country	Studied population	Equipment	Treatment group vs Controlled group (N)	Outcome	Major complication/Death
Pokushalov *et al*. (2012) [51]	Russia	Paroxysmal & persistent AF with drug-resistant HTN	Thermocool ablation catheter	PVI+RDN (13) vs PVI (14)	Freedom from AF at 1 year; 9 in PVI+RDN (69%) vs 4 in PVI (31%), *p* = 0.033	None
Pokushalov *et al*. (2014) [54]	USA	AF and HTN	Thermocool ablation catheter & Symplicity RDN catheter	PVI+RDN (41) vs PVI (39)	Freedom from AF at 1 year; 26 in PVI+RDN (63%) vs 15 in PVI (37%), *p* = 0.014	None
Kiuchi *et al*. (2016) [53]	Brazil	Paroxysmal & persistent AF with CKD & HTN	Irrigated ablation catheter	PVI+RDN (39) vs PVI (96)	Reduced AF recurrence in PVI+RDN group on F/U of 22.4 ± 12.1 months	None
Romanov *et al*. (2017) [55]	USA & Russia	Paroxysmal & persistent AF with drug-resistant HTN	Thermocool ablation catheter & Symplicity RDN catheter	PVI+RDN (39) vs PVI (37)	Freedom from any AF at 1 year; 0.61 risk reduction in PVI+RDN [95% CI: 0.51–0.81]	None
Kiuchi *et al*. (2018) [56]	Brazil	CKD patients with drug-refractory paroxysmal AF & HTN	EnligHTN RDN catheter	PVI+RDN (33) vs PVI (36)	(1) Freedom from AF at 1 year; 20 in PVI+RDN (61%) vs 13 in PVI (39%), *p* = 0.02. (2) Reduced mean AF burden in PVI+RDN after 12 mo; –12%, *p* < 0.0001	None
Steinberg *et al*. (2020) [52]	Poland, Russian, and Germany	Paroxysmal AF patients with hypertension	Irrigated ablation catheter	PVI+RDN (154) vs PVI (148)	Freedom from any atrial arrhythmia at 1 year; 111 in PVI+RDN (72%) vs 43 in PVI (28%), *p* = 0.006	1 MI & 2 cardiac/vascular surgery/2 (1.3%) death unrelated to the procedure
Turagam *et al*. (2021) [57]	USA	Drug-resistant paroxysmal & persistent AF	Thermocool ablation catheter	PVI+RDN (13) vs PVI (17)	No statistical significance found for AF freedom at 2 years F/U	renal artery stenosis (3/13) & renal artery dissection (3/13)
Turagam *et al*. (2021) [57]	USA, Czech Republic, Russia	Drug-resistant paroxysmal & persistent AF	Vessix RDN catheter	PVI+RDN (28) vs PVI (22)	No statistical significance found for AF freedom at 2 years F/U	None

AF, atrial fibrillation; CKD, chronic kidney disease; HTN, hypertension; PVI, pulmonary vein isolation; RDN, renal denervation; MI, myocardial infarction; F/U, follow up.

**Fig. 7.**
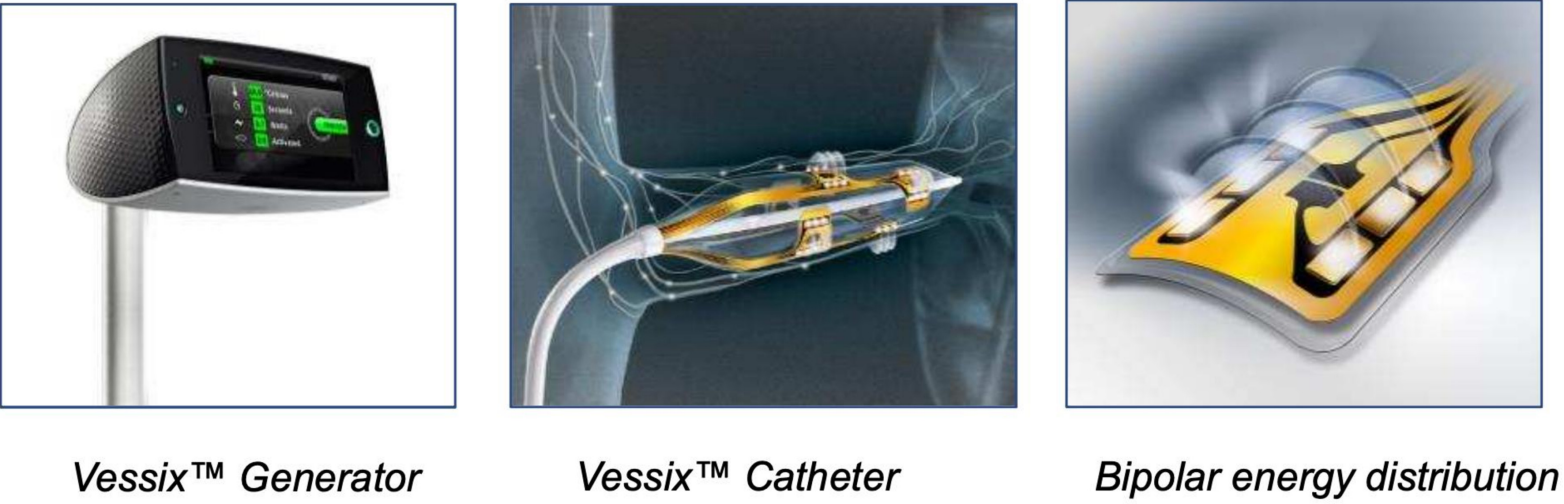
**Boston Scientific’s Vessix renal denervation system**. Over-the-wire angioplasty balloon catheter system (balloon diameters of 4, 5, 6, 7 mm) with thermistors and electrical gold contact on the exterior part. This system uses a bipolar RF energy. Simultaneous RF energy delivery of all electrodes with automatic deactivation on non-contact electrode. With courtesy from Boston Scientific. RF, radiofrequency.

(2) Boston Scientific VESSIX system (Fig. 7): The VESSIX system [58] features a balloon catheter with an array of RF electrodes arranged to cover the renal nerves distributed around the renal arterial wall. This catheter is designed to maximize the efficiency of RDN by offering a remarkably short treatment duration of only 30 seconds per artery. The interruption of the blood flow by an occlusion of the renal artery with the balloon allows for the direct and precise delivery of energy to the targeted nerves. Non-contact electrodes are deactivated, and the system employs a bipolar energy distribution to ensure accurate targeting with very low energy doses (≤1 Watt).

(3) Abbott/SJM enligHTN system [59]: The enligHTN system [60] comprises an 8F multi-electrode basket catheter with four evenly-spaced electrodes that is designed to deliver faster and more precise RF energy to the renal nerves. Each treatment session requires an average of 90 seconds, leading to fewer catheter positioning steps. However, it is noted that this catheter is currently no longer commercially available due to a discontinuation in the product pipeline.

## 4. Complications Related to RDN

Potential complications related to RDN procedures include, but are not limited to, arterial stenosis (0.3%) and arterial rupture [61]. Previous studies have shown no significant arterial stenosis at 6 months post-ablation with the Medtronic SYMPLICITY Spyral catheter [62, 63]. The location of the electrodes is sensitive to subtle motion, making it crucial to avoid patient movement during the procedure, even during respiration. The system incorporates a sensitive temperature detector that automatically aborts RF energy delivery if any issues arise, prompting the operator to reimaging of the renal artery to rule out an arterial spasm. In cases of a reduced blood flow due to spastic artery, nitroglycerin is administered to resolve this issue [64]. Ablation on diseased arterial structures, such as fibromuscular dysplasia, existing arterial stenosis, atherosclerosis, or aneurysm, is prohibited to mitigate a potential risk of the complications. According to a recent comprehensive meta-analysis, the combined complication rate in the PVI and RDN group, pooled from 7 clinical studies, was at 6.32%. The combined rate of complications in the PVI alone group was also unexpectedly high at 11.8%. Notably, this concerning statistic was primarily attributed to vascular access complications, which may or may not be directly associated with RDN’s technology or tool. It is essential to highlight that all instances of renal artery stenosis (3/13) and renal artery dissection (3/13) stemmed from the HFIB-1 study in this analysis, where the Thermocool ablation catheter was employed without Food and Drug Administration (FDA) approval for RDN [65].

## 5. Clinical Studies of RDN as an Adjunct Therapy to AF Ablation

The first clinical evidence of the benefits of RDN as an adjunct therapy to PVI came from Pokushalov’s study [51]. In this small randomized study, a freedom from AF at 12 months was significantly higher in the RDN+PVI group (9/13 patients, 69%) than the PVI-only group (4/14 patients, 29%) (*p* = 0.033). This exciting finding sparked an interest in several subsequent clinical studies investigating RDN’s role as an adjunct therapy to conventional PVI.

The Evaluate Renal Denervation in Addition to Catheter Ablation to Eliminate Atrial Fibrillation (ERADICATE AF) trial stands as a landmark trial that showcases the efficacy of RDN as an adjunct therapy to AF ablation [52]. In patients with poorly controlled hypertension and paroxysmal AF, the combination of RDN and AF ablation significantly improved an AF freedom at 1 year when compared with AF ablation without RDN (71.4% vs. 57.8%, hazard ratio of 0.61, confidence interval of 0.41–0.9, *p* = 0.011), with similar complication rates. Various small randomized controlled trials have also been conducted, showing favorable outcomes [37, 40, 66, 67]. A meta-analysis by Ukena *et al*. [68] included 689 AF patients with poorly controlled hypertension (those who failed to control blood pressure with three antihypertensive medications) from 5 out of the 6 included studies. The results of this meta-analysis demonstrated significant reduction of the blood pressure as well as AF recurrence (mean odds ratio of 0.43 with a 95% confidence interval) in patients treated with both RDN and PVI (either RF or cryoablation) compared to PVI alone. While the meta-analysis and its subsequent updates confirmed these observations, it is essential to consider the influence of the results derived from large randomized controlled trials like ERADICATE AF. Table 1 compiles essential randomized controlled trials pertinent to the outcomes of RDN in the context of AF suppression.

Several ongoing studies are currently adding more knowledge to this field. The Trial to Evaluate Renal Artery Denervation in Addition to Catheter Ablation to Eliminate Atrial Fibrillation (ERADICATE AF II) aims to test the hypothesis that combining PVI with RDN can provide a long-term antiarrhythmic effect compared to PVI alone for patients with symptomatic persistent AF without hypertension or with well-controlled hypertension. This multi-center, single-blinded, randomized controlled trial requires all subjects to have a loop recorder implanted for a precise AF burden calculation. The positive results of this study would have a potential to establish RDN as an adjunct therapy to AF ablation, with the possibility of becoming a standard care for persistent AF patients.

## 6. Unresolved Cardinal Questions

Indeed, the effectiveness and cost-effectiveness of RDN as a treatment approach for resistant hypertension have been a subject of numerous studies, and their mixed results lead to ongoing debates among scholars. Some studies have shown that RDN can be a cost-effective approach [69, 70], while others have not found any significant benefits in blood pressure reduction from RDN. One pivotal study, the SYMPLICITY-3 trial conducted in 2014 [71], demonstrated no significant benefit in blood pressure reduction from RDN. However, it is crucial to note that this trial used the first-generation ablation catheter with a single electrode, which did not allow for a verification of contact between the electrode and the arterial wall. Subsequent clinical studies utilizing the second-generation devices, such as the DENERHTN trial [72], the Spyral HTN-ON MED trial [73, 74], and the Spyral HTN-OFF MED trial [75, 76], have shown promising results with a significant blood pressure reduction. These second-generation devices have offered more advanced features and improvements in the ablation catheter technology.

The exact regimen of the ablation, including the number of lesions, distance between lesions, amount of RF energy delivery, and exposure time, has not been conclusively supported by previous studies. As a result, the therapeutic effect of RDN on a blood pressure control remains a topic of interest and feasibility for future researches. It is important to consider that the effectiveness of RDN may be influenced by factors such as the experience and skill of the practitioners performing the procedure and the careful patient selection. As more researches are conducted with advanced ablation catheter technologies and improved techniques, the potential benefits of RDN for resistant hypertension may become more evident and established.

### 6.1 What is the Anti-Arrhythmic Mechanism of RDN? And is It Durable?

The precise mechanism by which RDN exerts its effects to prevent or alleviate arrhythmia remains uncertain. The animal studies (Table 2, Ref. [25, 33, 43, 67, 77, 78]) have suggested that an independent action separate from its antihypertensive effect might operate as an antiarrhythmic mechanism [33, 45, 79]. Recent clinical research has corroborated this notion, revealing a significant reduction in subclinical AF in hypertensive heart disease patients who underwent RDN compared to those who received a sham procedure [31]. Importantly, there was no significant change in blood pressure between these two groups, thus demonstrating the pure anti-arrhythmic effect of RDN beyond its blood pressure-reducing or PVI benefits. Although the 2020 ESC guideline for the diagnosis and management of AF, developed in collaboration with the European Association for Cardio-Thoracic Surgery (EACTS), briefly acknowledges the role of RDN in supporting the benefits of blood pressure control in AF management [80], an expanding body of evidence suggests that the favorable effect of RDN in suppressing AF operates through mechanisms independent of blood pressure regulation.

**Table 2.** **Summarized key pre-clinical studies of RDN**.

Study (years)	Experimental model	Procedure	Outcome	Mechanistic insight
Zhao *et al*. (2012) [67]	Dogs with rapid atrial pacing	RDN group underwent RDN procedure vs control group	Persistent reduction of AERP was found in both groups. After 7 hrs of pacing termination, induced AF frequency and duration was higher in control group than RDN group. There was trend of reduced renin and aldostereone in RDN group	RDN reduced AF episodes during rapid atrial pacing with decreased activity of RAAS
Hou *et al*. (2013) [25]	Hypersympathetic tone canine model with left stellate ganglion stimulation and rapid atrial pacing	RDN group underwent RDN procedure while control group underwent sham procedure	RDN reversed AF induction rate, shortened ERP and increased ERP dispersion, and elevated plasma norepinephrine level compare to control group	RDN reduced AF inducibility and reversed atrial physiologic change from hypersympathetic activity
Linz *et al*. (2013) [77]	Normotensive anesthesized pig	All pigs underwent both procedures (renal denervation; RDN & baroreflex stimulation; BRS). HR, BP, atrial electrophysiology properties, and AF inducibility was measured	(1) Vagally-mediated shortened AERP leading to increased AF inducibility was observed after BRS, but not after RDN. (2) Shortened AERP was reversible after stopping BRS	RDN and BRS at the level of comparable BP and HR reduction influenced atrial electrophysiology differently. Increased vagal tone was found in BRS, but not in RDN, potentially caused shortened atrial refractoriness leading to increased AF inducibility
Linz *et al*. (2014) [33]	Goats with instrumented endocardial atrial lead and burst pacemaker	RDN group underwent RDN procedure while control group underwent sham procedure	RDN reduced sympathetic nerve which resulted in lower transcardiac norepinephrine levels. This was associated with less atrial nerve sprouting. Atrial endomysial fibrosis content was lower and myocyte diameter was smaller in RDN group. No significant BP difference observed between both groups	In goats with persistent AF, RDN reduced atrial sympathetic nerve sprouting, structural change, and AF complexity independent of BP change
Wei *et al*. (2016) [78]	Male New Zealand white rabbits	Abdominal aortic constriction (AAC) group vs sham-operated group vs RDN+AAC group. AF was induced by atrial pacing. Renin, angiotensin II, and aldosterone were measured	(1) AF inducibility rate was higher in AAC > AAC+RDN > sham-operated group. (2) AAC-induced elevation of collagen I, CTGF and TGF-β1. This elevation was suppressed by RDN	RDN suppressed the inducibility of AF in a model for pressure associated atrial fibrosis. The mechanism likely operated through modulating renin-angiotensin-aldosterone system and decreasing pro-fibrotic factors
Sharp *et al*. (2022) [43]	Normotensive Yorkshire farm swine	RDN was performed with RF energy and renal tissue samples were obtained after 7, 28, and 180 days. Renal cortical axon density and cortical NE level were measured. Scoring system was applied to downstream nerve fiber atrophy and tissue fibrosis	Axonal loss was present at the ablation site and its downstream at 7, 28, 180 days. Renal cortical axon density and cortical NE level were significantly reduced at 7 days in RDN group and it remained low at 180 days	Functional nerve growth after RDN utilizing RF energy is unlikely at 180 days post-procedure

AAC, abdominal aortic constriction; AERP, atrial effective refractory period; AF, atrial fibrillation; BP, blood pressure; BRS, baroreflex stimulation; HR, heart rate; NE, norepinephrine; RAAS, renin-angiotensin aldosterone system; RDN, renal denervation; RF, radiofrequency; ERP, effective refractory period; CTGF, connective tissue growth factor; TGF-β1, tissue growth factor-β1.

Emerging concepts in AF pathogenesis revolve around metabolic remodeling in cardiac tissues due to imbalances in sympathovagal activity [10, 11, 15, 81]. Sympathetic activity is believed to promote metabolic derangement, while cholinergic activation has the opposite effect. Animal models show that glycolytic inhibition is associated with elevated diastolic calcium, leading to frequent early after-depolarization firing from the pulmonary veins, ultimately precipitating acute AF episodes [3, 11, 12]. Moreover, imaging studies using Iodine-123 meta-iodobenzylguanidine scanning have confirmed the role of enhanced sympathetic activity in AF progression from a paroxysmal to persistent form. Conversely, the augmentation of parasympathetic activity via activated IKAch (acetylcholine-dependent activation of a cardiac potassium (K+) channel) can abbreviate the atrial refractory period. Adrenergic surges can increase intracellular calcium transients and shortened action potential duration. It is crucial to note that most phase-3 early after depolarizations are typically associated with prolonged action potential duration. However, the combined impact of sympathovagal activation can override repolarization from IKAch activation, inducing significant calcium transients. Consequently, it necessitates a collaborative role of both autonomic systems to induce late phase-3 early after depolarization, triggering arrhythmias. The heterogeneity in atrial refractory periods and action potential durations also facilitates the reentry mechanism for AF [11, 67, 82]. By mitigating the sympathetic effect, RDN contributes to favorable AF control outcomes in patients with a hyper-sympathetic state. However, as the role of the parasympathetic system in AF initiation and progression is more complex compared to the sympathetic system, RDN, which only addresses one side of this equilibrium, may not be sufficient for AF control in patients with more intricate pathophysiology. RDN has also been associated with a reduced heart rate, delayed atrioventricular (AV) node conduction time, or reduced ventricular rate during AF rhythm compared to beta-blocker use [10, 11, 33, 79]. In summary, the beneficial effects of RDN on AF control are multifactorial in nature.

The duration of RDN’s effects remains unknown and requires further investigation. The AFFORD (Atrial fibrillation reduction by renal sympathetic denervation) study observed durable anti-hypertensive efficacy for at least three years post-RDN without a change in AF burden [66]. However, limitations in the trial, such as the absence of a sham-control group and lack of statistical power to observe changes in AF burden, as well as the use of a clinical tool not officially approved for a clinical use (SJM EnligHTN system), warrant a cautious interpretation. Given that hypersympathetic tone promotes a structural remodeling in the atria, a durable effect from RDN should offer protective benefits against AF progression. Evidence indicates that a reduced sympathetic tone from neuromodulation may improve a left atrial function and mitigate left atrial fibrosis [11]. While theoretical possibilities of delayed compensatory reinnervation exist, histological studies showed no regrowth of the ablated nerves after 180 days, and injured nerves develop disorganized sprouting without any functionality [43, 83]. The interplay between the sympathetic tones from the aortorenal ganglion and other sympathetic systems, such as the left stellate ganglion and how these systems compensate or respond to RDN will ultimately dictate the long-term outcome of this treatment. Table 2 encapsulates pivotal animal studies that offer mechanistic insights into the efficacy of RDN in attenuating AF.

### 6.2 What are the Best Parameters to Verify the Adequacy of RDN? 

The lack of reliable parameters to assess a successful RDN is indeed a significant challenge. Having a specific test of renal sympathetic function that can provide immediate results would be highly beneficial in making decisions about the adequacy of the ablation during the procedure. Unlike a cardiac ablation, where local impedance drops during ablation can serve as indicators of success, such measures cannot be directly applied to RDN using RF energy. However, the absence of a significant impedance drop with RF application in RDN may prompt the need for catheter repositioning.

Recently, a proposed method involves using high-frequency pacing in the inferior vena cava and aorta to target the aortorenal ganglia [35]. This technique aims to observe a change in ipsilateral renal arterial vasoconstriction as the endpoint of RDN. In a large animal model, the location of the aortorenal ganglia has been successfully identified by using pacing map techniques at the junction of the renal artery originating from the abdominal aorta. After performing RDN, the abolition of aortorenal ganglion pacing-induced vasoconstriction observed on renal arterial angiogram could serve as an endpoint of the procedure. This method shows a promise and presents an interesting approach to assess the efficacy of RDN. It is anticipated that further research and testing in human subjects will be conducted to validate the feasibility and effectiveness of this technique, which could potentially provide a reliable parameter for evaluating a success of RDN in real-time during the procedure.

Various surrogate markers have been utilized to assess the autonomic effect [16], but they have not been extensively studied for RDN. These markers include heart rate variability, skin sympathetic nerve activity, direct muscle sympathetic nerve activity, or cardiac imaging techniques such as 123-iodine-metaiodobenzylguanidine or 11-carbon-meta-hydroxyephedrine to measure sympathetic activity. These methods may be beneficial in clinically observing the effect of RDN on the central sympathetic outflow during the follow-up period.

By incorporating additional methods to evaluate the effect of RDN, we can refine the protocol and tools for RDN procedures to achieve reproducible outcomes in patients with AF. An individualized approach to ablation, considering patient characteristics, variations in the renal artery anatomy, and the type of catheter used, will be required to optimize the efficacy of RDN. Further research is warranted to explore and validate the most suitable parameters for assessing a successful RDN and its long-term effects on sympathetic activity and AF control. Such a development could significantly enhance the precision and outcome of RDN procedures for AF and other conditions.

### 6.3 Which Patient Population is Most Suitable for RDN as an Adjunctive Therapy to AF Ablation?

The patient population that shows promise as a candidate for RDN as an effective modality for AF control includes those with chronic kidney disease (CKD). In CKD patients, AF is highly prevalent due to enhanced sympathetic tone and atrial remodeling, characterized by increased interstitial fibrosis, which promote AF initiation and maintenance. Animal models have demonstrated that RDN can reverse left atrial remodeling and its electrophysiological properties [39], such as a reduced left atrial (LA) conduction latency and conduction heterogeneity, independent of a renal function and antihypertensive effects. RDN may help ameliorate the development of an arrhythmogenic substrate in CKD patients.

A single-center prospective double-blind randomized trial involving 45 well-controlled blood pressure patients with stage 2–3 CKD and AF supported this hypothesis, revealing a lower AF recurrence in the PVI+RDN group compared to the PVI-only group [53]. Importantly, RDN was reported to have been safe and might have even mitigated a renal pathology, such as a reduction of proteinuria, without compromising or improving overall renal function. These findings allay concerns about a potential worsening of kidney function by RDN therapy in CKD patients.

Furthermore, conditions with an increased sympathetic tone, such as obstructive sleep apnea [41], heart failure, and myocardial ischemia/infarction, might present clinical contexts where RDN could demonstrate significant benefits in AF prevention and treatment. Overall, patients with CKD and other conditions characterized by an enhanced sympathetic tone and atrial remodeling may represent promising candidates for RDN as an effective modality for AF control. Further research and studies in these patient populations will contribute to a better understanding of the potential benefits of RDN in managing AF and related cardiovascular conditions.

## 7. Conclusions

In summary, RDN has shown promise as an effective therapeutic modality for AF treatment. While it is not yet a standard of care, RDN’s percutaneous nature and robust safety data have led to a growing number of clinical trials in different patient populations. These trials are worth monitoring for further insights. Continued research and data collection on RDN’s long-term effects on AF freedom and its application in various patient populations, including non-hypertensive patients, will help solidify its role as an adjunct therapy to PVI in AF ablation. With an increasing body of clinical evidence supporting the positive outcomes of RDN in various stages of AF across different patient populations, percutaneous catheter-based RDN emerges as a highly promising intervention to be conveniently administered during the same index procedure as standard pulmonary vein isolation by cardiac electrophysiologists.

## Abbreviations

AF, atrial fibrillation; ANS, autonomic nervous system; CKD, chronic kidney disease; PVI, pulmonary vein isolation; RDN, renal denervation; RF, radiofrequency.
